# Accelerometer-derived sleep metrics in adolescents reveal shared genetic influences with obesity and stress in a Brazilian birth cohort study

**DOI:** 10.1093/sleep/zsae256

**Published:** 2024-10-29

**Authors:** Marina Xavier Carpena, Karen Sanchez-Luquez, Mariana Otero Xavier, Ina S Santos, Alicia Matijasevich, Andrea Wendt, Inacio Crochemore-Silva, Luciana Tovo-Rodrigues

**Affiliations:** Postgraduate Program in Epidemiology, Federal University of Pelotas, Pelotas, RS, Brazil; Postgraduate Program in Epidemiology, Federal University of Pelotas, Pelotas, RS, Brazil; Departamento de Medicina Preventiva, Faculdade de Medicina FMUSP, Universidade de São Paulo, SP, Brasil; Postgraduate Program in Epidemiology, Federal University of Pelotas, Pelotas, RS, Brazil; Departamento de Medicina Preventiva, Faculdade de Medicina FMUSP, Universidade de São Paulo, SP, Brasil; Programa de Pós-Graduação em Tecnologia em Saúde, Pontifícia Universidade Católica do Paraná, Curitiba, Brazil; Postgraduate Program in Epidemiology, Federal University of Pelotas, Pelotas, RS, Brazil; Postgraduate Program in Epidemiology, Federal University of Pelotas, Pelotas, RS, Brazil

**Keywords:** actigraphy, stress, obesity, clock genes, genetic epidemiology, pediatrics–adolescents

## Abstract

We aimed to test the association between sleep-related polygenic scores (PGSs) and accelerometer-based sleep metrics among Brazilian adolescents and to evaluate potential mechanisms underlying the association through the enrichment of obesity, and cortisol pathway-specific polygenic scores (PRSet). Utilizing data from The 2004 Pelotas (Brazil) Birth Cohort, sleep time window and sleep efficiency were measured at the 11-year-old follow-up using ActiGraph accelerometers. Three sleep PGSs were developed based on the most recent genome-wide association study of accelerometer-based sleep measures. PRSet, calculated using variants linked to body mass index (BMI) and plasmatic cortisol concentration, aimed to assess pleiotropic effects. Linear regression models, adjusted for sex and the first 10 principal components of ancestry, were employed to explore the impact of sleep PGS and specific-PRSet on sleep phenotypes. The number of nocturnal sleep episodes-PGS was positively associated with sleep time window (β = 2.306, SE: 0.92, *p* = .011). Nocturnal sleep episodes were also associated with sleep time window when restricted to BMI-PRSet (β = 2.682, SE: 0.912, competitive *p* = .003). Both the number of sleep episodes and sleep time window cortisol-PRSets were associated (β = .002, SE: 0.001, *p* = .013; β = .003, SE: 0.001, *p* = .003, respectively) and exhibited enrichment in molecular pathways (competitive *p* = .011; competitive *p* = .003, respectively) with sleep efficiency. Sleep polygenetic components observed in European adults may partially explain the accelerometer-based sleep time window in Brazilian adolescents. Specific BMI molecular pathways strengthened the association between sleep PGS and sleep time window, while the cortisol concentration pathway had a significant impact on the genetic liability for sleep efficiency. Our results suggest genetic overlap as a potential etiological pathway for sleep-related comorbidities, emphasizing common genetic mechanisms.

## Graphical Abstract

**Graphical Abstract:**
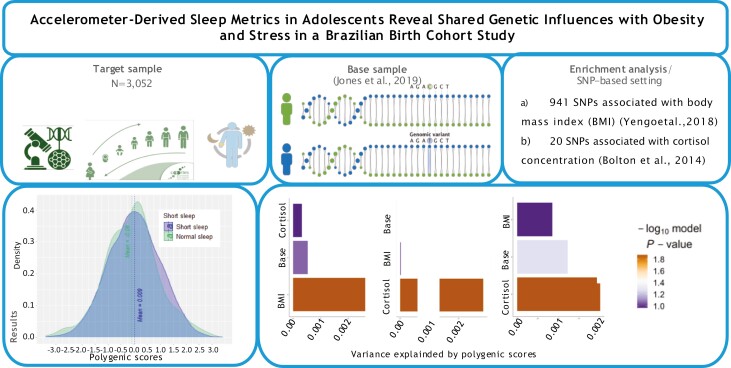

**Statement of Significance**

This study leverages polygenic scores (PGS) for sleep traits alongside body mass index (BMI) and cortisol-specific genetic pathways to examine polygenic components of accelerometer-delivered sleep metrics among Brazilian adolescents for The 2004 Pelotas Birth Cohort. Findings suggest that genetic factors impacting sleep in European adults may also play a role in Brazilian youth sleep duration, with BMI pathways associated with longer sleep durations and cortisol pathways influencing sleep efficiency. These insights reveal shared genetic mechanisms connecting sleep, obesity, and stress during adolescence, a crucial phase for neurodevelopment and mental health. The results underscore the importance of further exploring these genetic relationships across diverse populations.

Sleep is a complex phenomenon, essential for psychological and physical well-being. The current understanding of the sleep–wake rhythm is intricately linked to the internal circadian system, predominantly centered in the suprachiasmatic nucleus (SCN) within the anterior hypothalamus [1]. Disruption in the circadian rhythm resulting from insufficient or low-quality sleep can impact the secretion rhythm of crucial hormones including cortisol, harm the immune system, and potentially contribute to the development of metabolic diseases, such as obesity [2, 3].

Fundamental circadian genes, including *CLOCK*, *PER1/2*, and *CRY1/2*, form an elaborate network that orchestrates the circadian cycle. These genes are critical in regulating the sleep–wake cycle, modulating the release of hormones such as cortisol and melatonin, and governing fluctuations in body temperature, all of which are integral markers of circadian rhythm [4]. Expanding upon this knowledge, recent advancements have significantly enriched our understanding of polygenic influences on sleep phenotypes. The most comprehensive genome-wide association study (GWAS) on accelerometer-derived sleep measures to date, involving nearly 700 000 individuals, revealed additional loci not previously identified in self-report GWAS studies, uncovering 47 genetic associations [5]. A higher heritability for sleep duration was observed using accelerometer-derived measures compared to self-report measures, emphasizing the importance of considering various measurement methods to understand the heterogeneous findings in sleep research [5].

Several epidemiological studies conducted in diverse geographical regions suggest that short sleep duration and poor sleep quality are linked to an increased risk of obesity during childhood and adolescence [6, 7], as well as to altered cortisol levels [2, 8, 9]. These observations are further supported by genomic research, which reveals genetic correlations between complex human traits such as sleep, body mass index (BMI), cardiometabolic, and stress-related phenotypes [10, 11], and suggests that there is not only a genetic correlation but also a potential intrinsic causal relationship. Mendelian Randomization analysis provided substantial evidence for a potential causal effect of an increased waist-hip ratio on reduced sleep duration and decreased sleep efficiency [12].

Additionally, genes linked to stress reactivity, such as *BDNF* and *MC4R*, have been implicated in the interplay between sleep and BMI [10]. Current evidence points to shared biological pathways between sleep and cortisol concentrations, highlighting an emerging field of research that seeks to unravel the complex genetic interactions between these two areas [2, 13]. For instance, variations in genes responsible for cortisol’s production, transport, or metabolism can significantly affect its levels in the body, influencing sleep’s circadian rhythm, quality, and recovery processes. Furthermore, genetic factors affecting cortisol response play a critical role in stress management. Since stress can disrupt sleep patterns, genes that amplify stress sensitivity may also increase the propensity for sleep disturbances [13].

From these perspectives, studies exploring the connections between sleep and BMI, as well as between sleep and cortisol levels, have utilized diverse populations and methodologies. However, numerous questions remain unanswered in this field, which demands significant attention. Obesity rates [14], alongside the increasing prevalence of sleep disorders and circadian misalignment [15–17], have become alarming worldwide. Genetic epidemiology, particularly through polygenic score analyses, offers a valuable approach to uncovering shared genetic underpinnings among these traits. Mechanistic interactions and interconnections among brain regions implicated in gene expression across various GWAS on sleep suggest their potential role in regulating metabolism [10]. Furthermore, sleep disorders, including short sleep duration, act as chronic stressors that disrupt the normal functioning of the hypothalamic-pituitary-adrenal (HPA) axis. This axis governs cortisol production and release, impacting appetite-regulating hormones such as leptin and ghrelin, thereby contributing to an increased risk of obesity [18, 19].

Emerging research underscores the parallel evolution of sleep patterns and brain structure and activity, particularly during developmental phases, influencing long-term neurological outcomes [20, 21]. Adolescence is marked by dramatic shifts in neuroendocrine functions, behavior, and stress regulation, making it a pivotal period for the onset of mental health issues [22, 23]. Understanding the genetic variants that regulate sleep could provide important information about the mechanism by which sleep may act both as a risk factor or buffer in the process of human neurodevelopment, interfering with long-term brain structure and intervening in the onset of stress symptoms, mental disorders, and/or obesity throughout life. Research into the transferability of polygenic score (PGS) in adolescent populations of different genetic ancestrally backgrounds might also provide relevant and novel information on sleep-related traits.

In this study, our objective was to investigate the association between PGS for sleep-related phenotypes and accelerometer-derived sleep metrics among adolescents from the 2004 Pelotas (Brazil) Birth Cohort. Additionally, the correlations among sleep, BMI, and cortisol levels reported in the literature may be partly attributable to pleiotropy, a phenomenon where a single genetic locus influences multiple, seemingly unrelated phenotypic traits [24, 25]. Consequently, we explored pathway-specific PGSs (PRSets), utilizing BMI and cortisol single nucleotide polymorphisms (SNPs)-related, to further elucidate the complex interconnections among these variables.

## Methods

### Data sources

The data utilized in this research were derived from the 2004 Pelotas Birth Cohort, a population-based longitudinal study initiated in 2004. This study involved recruiting newborns from the five maternity hospitals in Pelotas, a city in southern Brazil. Initially, the cohort encompassed 4231 infants born to mothers residing in the urban area of the city. These participants were subsequently followed at multiple stages throughout their childhood and adolescence. The follow-up assessments occurred at various intervals: at birth (with a 99.2% retention rate), 3 months (95.7% retention), 12 months (94.3% retention), 24 months (93.5% retention), 48 months (92.0% retention), 6 years (90.2% retention), 11 years (86.6% retention), and finally at 18 years of age (85.0% retention). The 2004 Pelotas Birth Cohort was designed to evaluate various social, economic, and health factors among mothers and participants. Therefore, its scope is not limited to assessing sleep behavior alone. For the purposes of this study, we specifically utilized data from the perinatal period and the 6- and 11-year follow-up intervals. Further details regarding the original study’s design and methodology are documented in previous publications [26–28].

### Sleep phenotypes

At 11 years of age, 3566 adolescents participated using accelerometer devices to monitor human activity and rest cycles. The ActiGraph accelerometer, model wGT3X-BT (ActiGraph, Pensacola, FL), was fixed to the participant’s non-dominant wrist to collect sleep information. Participants were instructed to use the accelerometer 24 hours a day, 7 consecutive days, reaching 6 days with complete information. We analyzed all participants who had at least three nights of assessed data with valid results. The device monitors the period of sleep and wakefulness, recording body movement (and absence of, or very limited movement) day and night. Accelerometer was delivered to the participants in the end of their visit to the research clinic by trained members of the research team, and collected later in the participants’ house on a scheduled day/time. Data were downloaded daily using the device-specific software (ActiLife) and stored in raw data format, with weekly backups. The accelerometer raw data underwent processing the GGIR package in R software [29], which included several critical steps: autocalibration, non-wear detection, and data cleaning [30]. Additionally, automatic sleep detection was performed using the GGIR package [29]. Previous studies have established the validity of the GGIR algorithm in adolescents [31], supporting the reliability of this study. In brief, the sleep detection algorithm first identified sleep windows, which are characterized by lower sustained acceleration levels. Subsequently, the algorithm searched for 5-minute windows during which there were minimal changes (<3°) in wrist angle. These windows were designed to overlap, allowing the algorithm to identify periods of sleep based on the absence of significant changes in wrist angle, distinguishing them from periods of wakefulness.

We used two measures in our analysis:

1. Sleep time window (analogous to sleep duration): This refers to the time between sleep onset and sleep end within a 24-hour cycle, expressed in minutes.
2. Sleep efficiency: Calculated as the percentage of time spent sleeping within the sleep time window. Specifically, it is obtained by dividing total sleep time by the sleep time window, then multiplying by 100. Total sleep time is defined as the number of minutes during which wrist angle changes were less than 3° in 5-minute intervals, based on the algorithm by Van Hees et al. Sleep efficiency is expressed as a percentage.

We considered data valid if the sleep time window was between 3 and 18 hours. Any outliers falling outside this range were excluded from the analysis.

### Genotyping

At the 6-year follow-up in 2011, saliva samples were collected from 3722 participants of the cohort using the Oragene GenotekVR—kit 250.The genomic DNA was extracted from these saliva samples in accordance with the manufacturer’s guidelines. The DNA samples underwent genotyping via the Infinium Global Screening Array v.2 (Illumina). For those genetic variants not directly genotyped, imputation was carried out using SHAPEIT 2 [32] and MINIMAC3 [33] employing the global population data from phase 3 of the 1000 Genomes Project as a reference panel.

Stringent quality controls were applied: Only SNPs meeting specific criteria were included in the analysis. These criteria were: (i) less than 2% missing genotypes, (ii) an imputation quality score (R²) greater than .3, and (iii) a minor allele frequency of at least 0.01. This filtering process was conducted using PLINK 1.9 [34]. Additionally, any variants that deviated significantly from the Hardy–Weinberg Equilibrium, with a *p*-value less than 1e−6, were excluded prior to the construction of the score. After these rigorous quality control measures, a total of 11 811 746 genetic markers were available for analysis from 3472 individuals in the cohort.

### Covariables

Biological sex (male or female), prematurity (gestational age < 37 weeks), maternal smoking during pregnancy (yes or no), and total family income (in BRL, Brazilian currency) were used to describe our sample. These participant characteristics were assessed at the perinatal study (baseline). Additionally, we used mother-reported skin color of the child (categorized as white, non-white Brazilians), collected at the 6-year follow-up. Covariates such as the first 10 genomic principal components, estimated using the program PLINK1.9 [34], and biological sex were employed to adjust models for confounding factors like population stratification bias.

### Sleep-polygenic scores

For the construction of our PGS, we utilized summary statistics from the most comprehensive and recent GWAS that focused on accelerometer-based objective measures of sleep, including sleep duration, sleep efficiency, and the number of sleep episodes [5], which analyzed data from participants of European descent in the UK Biobank (UKBB) cohort. The UKBB is a national, prospective cohort study that enrolled over 500 000 men and women from various regions of the United Kingdom, aged between 40 and 69 years, during the period from 2006 to 2010. Specifically, the study by Jones et al. included 85 502 participants for sleep efficiency, 85 068 for sleep duration, and 85 502 for the number of sleep episodes. In the original GWAS, episodes were defined as periods of at least 5 minutes with minimal movement detected by the activity monitor, with the summed duration providing the overall sleep duration within a specified time frame [12]. Further details about the GWAS referenced in our research are available in Supplementary Table 1. The PGS for each participant in our cohort was calculated by summing the alleles associated with each sleep phenotype, using a *p*-value threshold (pT) of 1. We selected this threshold because it demonstrated the strongest association with the outcomes in our dataset, as detailed in Supplementary Table 2. Additionally, these alleles were weighted according to their effect sizes derived from the summary statistics, ensuring that the contribution of each allele reflected its relative influence on the phenotype. This calculation was performed using PRSice 2.0 software [35]. The resultant PGS was then standardized into z-scores, a step crucial for facilitating statistical model fitting. We presented the ΔR², representing the increase in R² with the addition of the PGS to the model in percentage (0.00%–100.00%), to isolate the explanatory power of the PGS from the included covariates, as recommended elsewhere [36].

### PGS enrichment set analysis

The SNP set consisted of SNPs selected based on their association with a specific trait or characteristic (e.g., cortisol or BMI). Enrichment analyses were conducted using a statistical test to determine whether the effect of these SNPs was significantly higher compared to the standard. The enrichment analyses were based on the following SNP set PGSs: (a) BMI_SNPs_Set: 941 near-independent SNPs associated with BMI in the most recent GWAS meta-analysis (significance threshold of *p* < 1 × 10^−8^, *N* = 700 000) [37]; (b) Cortisol_SNPs_Set: 20 single nucleotide polymorphism (SNPs) with annotation on *SERPINA6* and *SERPINA1* genes, previously reported by Bolton et al. [38]. Additional information about the SNPs included in each set can be found in Supplementary Table 3.

### Statistical analysis

Firstly, we described and compared the included sample with the original perinatal participants using the bivariate qui-square test. To assess the association between the PGS and sleep phenotypes, we used linear regression models adjusted for sex and the first 10 principal components of ancestry. The effectiveness of the PGS in predicting sleep phenotypes was determined by the incremental R-squared (ΔR²), which was calculated as the difference in R-squared values between the full model (incorporating all covariates, including PGS) and the null model (excluding PGS) as described before [39].

The initial step involved creating the ten principal components using PLINK 1.9 software and constructing the PGS with PRSice 2.0 [40]. Subsequent data analysis was conducted in R version 4.2.2. We employed the “stats” package for two main purposes: standardizing the PGS into z-scores and performing all statistical analyses. The regression results were expressed in terms of the Beta coefficient (β). For PRSet enrichment analyses, we used a competitive *p*-value to indicate the level of enrichment whereas a *p*-value was used to express the significance of association analysis. This approach contrasts with the self-contained *p*-value, which signifies the degree of association, as explained by Lee et al. Additionally, we applied the false discovery rate (FDR) *p*-value correction to account for multiple testing. Results were deemed statistically significant at *p* < .05.

### Ethical considerations

All follow-up phases of the 2004 Pelotas Birth Cohort were approved by the Research Ethics Committee of the School of Medicine of the Federal University of Pelotas. The genomic studies were also approved by the National Research Ethics Committee (CONEP). All mothers or guardians of participating children signed an informed consent form before data collection in both perinatal, 6 and 11 years old, and adolescents signed a consent form at 11 years.

## Results

### Sample characteristics

In this study, we included 3052 individuals for whom both genetic and sleep phenotype data were available (73.84% retention ratio). Table 1 presents detailed characteristics of the participants, comparing the included sample (*N* = 3052) with the original cohort (*N* = 4231). In the sample analyzed, approximately half (50.7%) were male. A significant proportion, 67.1%, identified as white Brazilians. Premature births were reported in 13.3% of cases, and around 27% of the participants were exposed to smoking during pregnancy. Regarding socioeconomic status, there were no differences in family monthly income between the included sample and the total Cohort sample (*p* = .3736). The included sample reported an average income of approximately BRL 794 per month, whereas the non-included sample reported around BRL 803. The average sleep time window among participants was 8.13 hours (standard deviation, SD: 0.83 hours), and the mean sleep efficiency was 85% (SD: 0.09%). We found no statistically significant differences in the comparative analysis between the group included in the study and the total cohort.

**Table 1.** Description of the sample according to prenatal, perinatal and sleep variables, and comparison between those included and the original cohort

Variables	Categories	Included (*N* = 3052)	Total cohort (*N* = 4231)	*p*
Biological sex (%)	Male	1546 (50.7)	2195 (51.9)	.307
	Female	1506 (49.3)	2036 (48.1)	
Skin color (%)	White Brazilian	2048 (67.1)	2726 (68.2)	.355
	Non-white Brazilians	1003 (32.9)	1272 (31.8)	
Prematurity (<37 weeks) (%)	No	2644 (86.7)	3603 (85.5)	.150
	Yes	406 (13.3)	612 (14.5)	
Smoking during pregnancy (%)	No	2238 (73.3)	3067 (72.5)	.153
	Yes	813 (26.7)	1162 (27.5)	
Family income at birth in BRL (SD)		794.16 (1087.27)	803.59 (1109.06)	.3736
Sleep time window at age 11 in minutes (SD)		488(49.7)	NA	NA
Sleep efficiency at age 11 in % (SD)		0.85 (0.0009)	NA	NA

2004 Pelotas Birth Cohort, Brazil.

### Association between PGSs and sleep phenotypes at 11 years

Table 2 presents the associations between the three constructed PGSs and sleep time window and sleep efficiency phenotypes. The genome-wide PGSs (GWAS-PGSs), developed using summary statistics for the number of sleep episodes, showed a significant association with increased sleep time window at age 11. Specifically, after adjusting for covariates, each 1 SD increase in the number of sleep episodes-PGS was associated with a mean increase of 2.31 minutes of sleep time window (SE = 0.92; *p* = .011). However, following adjustment for multiple testing, the statistical significance was not sustained (FDRp = .066). None of the other PGSs, based on sleep time window and efficiency summary statistics, were associated with either sleep time window or sleep efficiency.

**Table 2.** Results of the adjusted linear regression models for the association between genetic (PGSs) constructed as based on Jones et al. study [8] and sleep phenotypes (sleep time window, and sleep efficiency) among adolescents aged 11 (*N* = 3.052)

Exposure variables	Outcome phenotype	N SNPs	β	Standard error	*p*	FDR *p*	ΔR^2^ (%)
*GW-PGS*							
Sleep duration (Jones et al. [12])	Sleep time window	335 045	0.58	1.15	.609	.914	0.01
	Sleep efficiency		−0.0001	0.001	.960	.960	<0.01
Sleep efficiency (Jones et al. [12])	Sleep time window	307 735	−0.841	0.97	.386	.772	0.02
	Sleep efficiency		−0.0001	0.001	.954	.960	<0.01
Number of sleep episodes (Jones et al. [12])	Sleep time window	307 477	2.306	0.92	**.011**	.066	0.21
	Sleep efficiency		−0.0015	0.001	.088	.264	0.09

Pelotas Birth Cohort 2004, Brazil.

Sleep time window was regressed in minutes and sleep efficiency as % (dependent variables). Linear regression models were adjusted for sex and the first 10 ancestry-informative genetic principal components as covariates. N SNPs shows how many SNPs are included in each PGS. ΔR^2^ (%) shows the variance explained by the PGS, ranging from 0.00% to 100.00%. FDR p represented the false discovery rate corrected *p*-value. Statistically significant results are highlighted in bold.

**Figure 1.**
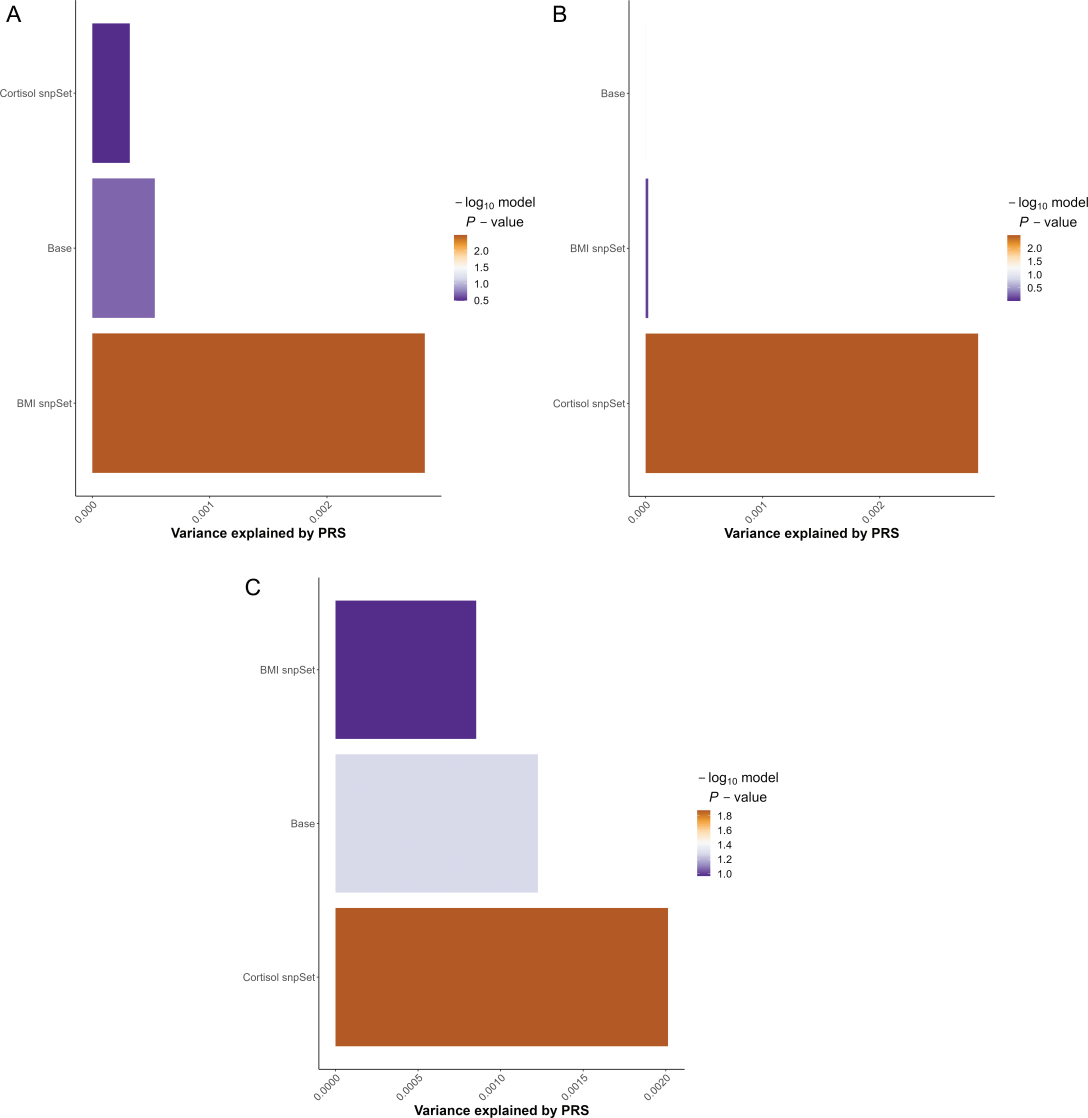
Multi-set plotting significant results of adjusted linear regression models for the association between pathway-specific polygenic scores (PRSet) and sleep phenotypes. (a) the association between pathway-specific sleep efficiency PGSs and sleep time window; (b) the association between pathway-specific sleep time window PGSs and sleep efficiency; (c) the association between pathway-specific number of sleep episodes PGSs and sleep efficiency.

The results of the enrichment analysis for the PRSets are presented in Table 3. The BMI-PRSet based on sleep efficiency summary statistics was positively associated with an increased sleep time window (β = 2.682; SE = 0.912; *p* = .003; FDR *p*-value = 0.018). This BMI-PRSet also retained a significant competitive *p*-value in the enrichment analysis following FDR correction (FDRp = .018), as illustrated in Figure 1.

**Table 3.** Results of the enrichment analysis for the PRSets constructed based on Jones et al. study [8], adjusted linear regression models among adolescents aged 11 (*N* = 3.052)

Pathway-specific set/ N SNPs	Jones et al. base data	Outcome Phenotype	β	Standard error	*p*	FDR *p*-value	ΔR^2^ (%)	Competitive *p*-value	FDR competitive *p*-value
BMI 734	Sleep duration	Sleep time window	0.994	0.912	.276	.414	0.04	.29**3**	.440
	Sleep efficiency		2.682	0.912	**.003**	**.018**	0.28	**.003**	**.018**
	Number of sleep episodes		−1.072	0.940	.253	.414	0.04	.254	.439
	Sleep duration	Sleep efficiency	0.0002	0.001	.794	.794	0.002	.798	.798
	Sleep efficiency		−0.001	0.001	.436	.516	0.02	.443	.532
	Number of sleep episodes*		−0.001	0.001	.107	.257	0.08	.107	.321
Cortisol 2	Sleep duration	Sleep time window	−0.645	0.898	.473	.473	0.02	.477	.477
	Sleep efficiency		−0.895	0.907	.324	.389	0.03	.339	.407
	Number of sleep episodes		−1.126	0.907	.214	.321	0.05	.214	.3210
	Sleep duration	Sleep efficiency	0.003	0.001	**.003**	**.018**	0.28	**.003**	**.018**
	Sleep efficiency		0.001	0.001	.099	.198	0.09	.100	.200
	Number of sleep episodes		0.002	0.001	**.013**	**.039**	0.20	**.011**	**.033**

Pelotas Birth Cohort 2004, Brazil.

*Number of SNPs included = 736; sleep time window was regressed in minutes and sleep efficiency as % (dependent variables). Linear regression models were adjusted for sex and the first ten ancestry-informative genetic principal components as covariates. N of SNPs shows how many SNPs are included in each PGS. ΔR^2^ (%) shows the variance explained by the PGS, ranging from 0.00% to 100.00%. Competitive *p* shows the *p*-value enrichment after 10 000 permutations in Analysis of enrichment. FDR p represented the false discovery rate corrected *p*-value. Statistically significant results In bold are highlighted in bold.

The cortisol-PRSet created using GWAS summary statistics for the sleep time window demonstrated a positive association with sleep efficiency (β = .003; SE = 0.001; *p* = .003), and this association persisted after correction multiple tests (FDRp = 0.018). Additionally, this sleep time window cortisol-PRSet exhibited a significant competitive *p*-value (*p* = .003, FDRp = .018) in the enrichment analysis.

Furthermore, the cortisol-PRSet for the number of sleep episodes also showed an association with increased sleep efficiency (β = .002; SE = 0.001; *p* = .013), and an enrichment effect (*p* = .011). Both of these associations remained significant after correction for multiple testing (association FDRp = .039; competitive FDRp = .033).

## Discussion

In this study, we used a unique combination of genetic and actigraphy data from a representative birth cohort to report for the first time the evaluation of the potentially polygenic and pleiotropic effects on sleep among Brazilian adolescents. Our results are consistent with previously identified molecular mechanisms identified in accelerometer-derived sleep measures in European adult populations. The findings suggest that polygenic components, previously documented in relation to adult number of sleep episodes, may partially account for sleep time window variations observed in Brazilian adolescents. Additionally, we have documented a consistent enrichment effect of *SERPINA6/SERPINA1* variants on sleep efficiency, as well as an association between sleep PGS created with BMI-related genetic markers and sleep time window. These findings underscore potential underlying mechanisms influencing sleep traits in adolescents.

We observed a suggestive positive association between the PGS for the number of sleep episodes and sleep time window. This finding is in line with previous research demonstrating the polygenic influence on sleep behavior [41, 42], although it explained a relatively small amount of variance. While circadian genes are well-established in the literature as central to the molecular system regulating sleep, recent discoveries have highlighted additional genes involved in the broader regulatory cascades of circadian rhythms, thus emphasizing the highly polygenic nature of sleep phenotypes [12, 41, 43]. Our findings are also supported by previous findings [41], which identified the effects of polygenic scores (PGS) on sleep time window in children and adolescents, using a GWAS based on self-reported sleep time window as the reference sample.

Our results revealed a major effect from SNPs associated with the number of sleep episodes, with no significant associations found for SNPs related to accelerometer-based sleep time window or sleep efficiency. Sleep episodes may reflect fundamental aspects of sleep regulation and fragmentation, which are pertinent to understanding genetic influences on sleep behavior. The shared genetic factors observed are consistent with findings from other studies on sleep traits. For instance, a recent GWAS reported an unexpected positive genetic correlation between short and long sleep time windows [44]. Additionally, findings by Kocevska et al. suggested that PGS for longer reported sleep duration predicted wake after sleep onset better than PGS for sleep duration. The observed cross-trait association in our study suggests that genetic factors influencing the number of sleep episodes may influence sleep time window metrics in the 2004 Pelotas Birth Cohort, posing challenges in disentangling the effects of specific variants uniquely associated with individual sleep traits [10]. Although some of our findings did not achieve statistical significance after multiple testing corrections, they contribute to the expanding evidence emphasizing the intricate and highly polygenic nature of sleep. By dissecting these complex relationships, researchers can better elucidate the genetic architecture underlying different sleep traits.

We utilized genetic pathway-based analyses to further explore the genetic overlap between sleep and BMI. Our results support previous findings indicating common genetic mechanisms linking sleep and weight [10, 45], as well as identifying specific genes that overlap between these traits [46]. Our study significantly contributes by demonstrating that a sleep PGS derived specifically from BMI-associated SNPs was associated with sleep time window among Brazilian adolescents. Additionally, we identified a BMI signaling pathway associated with these findings. Previous research by Garfield et al. indicated a non-significant genetic correlation between BMI and sleep time window, despite observing a slight association of a BMI-PGS with decreased self-reported sleep duration in a large cohort of older adults [47]. Our findings are particularly noteworthy as they reveal a stronger effect of the BMI-specific sleep PGS on the sleep time window compared to classical PGS effects observed in our study. By employing pathway-based PGS analysis, we enhanced the specificity of our investigation to determine whether BMI-related pathways are over-represented in the genome and associated with sleep phenotypes. The results suggest that focusing the sleep PGS on BMI-related SNPs may improve its predictive accuracy for the sleep time window. However, the identification of shared genetic influences between sleep and BMI can vary among different populations, particularly across different age groups. Evidence shows that genetic influences on BMI tend to increase during childhood, peak in early adulthood, and decline with advancing age, mirroring the decrease observed in the association between BMI and sleep time window over the lifespan [48]. Therefore, while our study presents promising findings, future research should investigate whether the enhanced effect of the sleep PGS using BMI-related genes remains consistent into adulthood and old age.

In our study, we observed an emerging sleep PGS effect on sleep efficiency when restricted to cortisol-SNPs which also showed significant pathway enrichment in the phenotype. Heightened stress reactivity is a well-established risk factor or correlate of sleep disturbances and insomnia [2, 49, 50]. Yap et al. recently emphasized the bidirectional relationship between perceived stress and sleep disturbances. Sleep disruption can heighten stress by activating the HPA axis, leading to dysregulated cortisol production, a key adrenal stress hormone [2]. Frequently used as a biomarker for HPA axis dysregulation, cortisol is linked to poor sleep and fatigue [51], and is implicated in regulating sleep quality [52]. The SNPs included in our cortisol set were located in *SERPINA6/1* genes. While the *SERPINA6* gene encodes corticosteroid binding globulin (CBG, the major cortisol-binding protein in plasma), *SERPINA1* encodes for α1-antitrypsin (which inhibits cleavage of the reactive center loop releasing cortisol from CBG) [38]. Then, these genes are specially important to modulation of the bioavailability of the cortisol molecule, which might suggest the molecular pathway in which *SERPINA1/6* might affect sleep efficiency. Then, these are important genes that should be prioritized for further functional studies not only in the sleep-stress context but specially to understand sleep efficiency phenotypic manifestations.

Our findings are specially important considering that this is the first study evaluating polygenic and pleiotropic effects on accelerometer-based sleep metrics among Brazilian adolescent participants from a population-based birth cohort study. Another strength is evaluating the participants during adolescence. The data obtained from this particular timeframe can yield novel and crucial findings [48]. However, our results might be interpreted in light of some limitations. The first concerns the genetic admixture of the Pelotas population. The base sample for PGS construction was derived from European populations. Although our analyses were adjusted for population stratification, the transferability of PGSs to our sample might have been impacted due to genetic differences. Secondly, we may be underestimating the effect of sleep PGS due to the difference in the genetic architecture of sleep duration between children and adults [53]. Therefore, our findings might not fully capture the nuances in adolescents since we constructed a sleep PGS with summary statistics derived from adults. Currently, there is no GWAS evaluating objective sleep measures in adolescents, making it unfeasible to test whether the effects would be greater or different if adolescent data were used as the base sample. While our study benefited from actigraphy assessment, the absence of sleep diaries represents a limitation in capturing self-reported sleep-related information or even validating accelerometer-base sleep measures. Furthermore, it is important to note that the use of an automatic sleep detection algorithm precludes the identification of the latency period, and the sleep time window does not account for periods of wakefulness within it. Finally, our study lacks predictive power, primarily due to the small fraction of variance explained. Nonetheless, we are keen on exploring genetic associations in a diverse population, thereby addressing an emerging need in the literature to enhance our understanding of complex and polygenic traits [36]. Thus, we highlight the need for further research in this area, particularly using diverse samples, and focusing on adolescent-specific sleep patterns and their genetic underpinnings, also including other sleep measures. Nevertheless, we should not overlook the genetic underpinnings identified for complex traits, which may also result from high genetic and environmental correlations. This underscores the importance of integrating broader environmental and lifestyle data to fully comprehend the intricate relationships between complex traits. In this regard, new approaches reported in the literature may prove beneficial, particularly in statistical modeling that incorporates these effects, such as genetically structured equation models [54].

While the polygenetic basis of sleep, BMI, and cortisol remains an active area of research, necessitating further exploration for a more comprehensive understanding of their intricate connections, our results suggest genetic overlap as a potentially specific etiological pathway for sleep-related comorbidities. Taking all together, we can consider the importance of more studies evaluating the complex relationship between sleep, BMI and stress from a perspective that takes into account the pleiotropism of the genes involved in different systems and traits. Our results, therefore, contribute to a growing body of evidence suggesting that the genetics of sleep, cortisol regulation, and BMI are interrelated. This knowledge could eventually lead to more targeted and effective interventions for sleep-related disorders and their associated comorbidities.

## Supplementary material

Supplementary material is available at *SLEEP* online.

zsae256_suppl_Supplementary_Tables_S1-S3

## Data Availability

Applications to use the data can be made by contacting the researchers of the 2004 Pelotas Birth Cohort (see https://www.epidemio-ufpel.org.br/site/content/faculty/ for a list of key faculty members) and completing the application form (https://www.epidemio-ufpel.org.br/site/content/studies/formularios.php). A list of administered questionnaires at each timepoint can be accessed online (https://www.epidemio-ufpel.org.br/site/content/coorte_2004-en/questionnaires.php). Researchers with successful applications will receive a dataset including the requested variables and unique participant IDs.
